# Theta and Alpha Oscillation Impairments in Autistic Spectrum Disorder Reflect Working Memory Deficit

**DOI:** 10.1038/s41598-017-14744-8

**Published:** 2017-10-30

**Authors:** Josefina Larrain-Valenzuela, Francisco Zamorano, Patricia Soto-Icaza, Ximena Carrasco, Claudia Herrera, Francisca Daiber, Francisco Aboitiz, Pablo Billeke

**Affiliations:** 1 División de Neurociencia, Centro de Investigación en Complejidad Social (neuroCICS), Universidad del Desarrollo, Av. Las Condes 12461, Las Condes, Santiago, 7590943 Chile; 2 Unidad de Imágenes Cuantitativas Avanzadas, Departamento de Imágenes, Clínica Alemana de Santiago, Av. Vitacura 5951, Vitacura, 7650568 Chile; 3 Laboratorio de Neurociencias Cognitivas, Departamento de Psiquiatría, Centro Interdisciplinario de Neurociencia, Pontificia Universidad Católica de Chile, Marcoleta 391, Santiago, 8330024 Chile; 4 Sociedad de Psiquiatría y Neurología de la Infancia y Adolescencia de Chile, Esmeralda 678, Santiago, 8320053 Chile

## Abstract

A dysfunction in the excitatory–inhibitory (E/I) coordination in neuronal assembly has been proposed as a possible neurobiological mechanism of Autistic Spectrum Disorder (ASD). However, the potential impact of this mechanism in cognitive performance is not fully explored. Since the main consequence of E/I dysfunction is an impairment in oscillatory activity and its underlying cognitive computations, we assessed the electroencephalographic activity of ASD and typically developing (TD) subjects during a working-memory task. We found that ASD subjects committed more errors than TD subjects. Moreover, TD subjects demonstrated a parametric modulation in the power of alpha and theta band while ASD subjects did not demonstrate significant modulations. The preceding leads to significant differences between the groups in both the alpha power placed on the occipital cortex and the theta power placed on the left premotor and the right prefrontal cortex. The impaired theta modulation correlated with autistic symptoms. The results indicated that ASD may present an alteration in the recruitment of the oscillatory activity during working-memory, and this alteration could be related to the physiopathology of the disorder.

## Introduction

Autistic spectrum disorder (ASD) is a severe and heterogeneous neurodevelopmental disease whose key features are social interaction impairments and the presence of restricted, repetitive patterns of both interest and behavior. Although there are some theoretical frameworks that explain the cognitive and social deficits, few links have been carried out in order to connect these deficits to neurobiological mechanisms. In this context, an influential hypothesis of the neurobiological mechanisms underlying ASD is a dysfunction in the excitatory–inhibitory (E/I) coordination in neuronal assembly^1,2^. The evidence supporting this hypothesis comes from converging clinical and animal model findings^3^. For example, clinical observations indicate that there exist both an increase prevalence of ASD in epilepsy and vice versa^4^, and an altered sleep EEG in children with ASD^1^. Since inhibitory interneurons are responsible, in part, for the coordination of neuronal oscillatory activity and rhythmogenesis^5^, the proposed inhibitory alteration in ASD may lead to impairments in neuronal oscillatory activity and synchrony. In fact, optogenetic animal models show that a reduction in E/I coordination generates an alteration in the information processing together with a reduction of the power of oscillatory brain activity^6^. This alteration in the oscillatory activity correlated with in social behavior deficits^7^. Indeed, studies in humans have shown that impairments in long-range neural synchronization could be a neuronal marker of ASD^8^.

Since both local oscillatory activity of neuronal assembly and long-range neuronal synchronization are key features of brain computation^5,9–11^, alterations in those mechanisms could lead to symptoms demonstrated in neuropsychiatric diseases like ASD^12–14^. Several studies using either magneto or electroencephalogram (EEG) have assessed for possible oscillatory activity alterations in patients with ASD. The most reported finding is an increase in the oscillatory power of several frequency ranges at rest^15–17^. By contrast, studies assessing long-range neural synchronization (i.e., inter-cite-synchrony) have found contradictory evidence. During rest, while some studies found an increase in neuronal synchrony^18,19^, others found a decrease^20^. There is little evidence of oscillatory activity related to cognitive processes in ASD that could reflect possible alterations in neural computation. For example, in ASD, the binding of the different features necessary for perceiving an object as a whole is altered. This alteration is related to a reduced oscillatory activity in both gamma and beta bands^21–24^. Similar results have been shown for motor control, where subjects with ASD present less oscillatory modulation in alpha and beta bands^25^. In this context, the study of working-memory (WM) is particularly relevant because it is a well-known cognitive process where oscillatory brain activity has a crucial role and a part of the key aspect of the psychopathology of several neuropsychiatric diseases.

In a healthy subject, an increase in memory load has been related to a particular change in oscillatory activity. On the one hand, an increase in alpha oscillations in occipital and posterior parietal regions has been interpreted as a deactivation of the sensory cortex during the delay period^26–28^. In fact, using simultaneous functional magnetic resonance imaging (fMRI) and EEG recordings, the alpha increase correlated with a BOLD signal decrease^29^. Nonetheless, in addition to the filtering of distractors, the modulation of alpha band may have a direct role in memory maintenance^30^. On the other hand, theta oscillations also participate during WM processes^31^. Frontal and parietal theta activity increases with memory load, and correlates with a BOLD signal increase in the premotor inferior frontal gyros^29,32^. Recently, the casual role of theta oscillations in WM has been demonstrated using rhythmic transcranial magnetic stimulation^33^. Therefore, WM paradigms may serve as a good approach to study oscillatory activity and its underlying computational function in ASD. Moreover, it has been proposed that executive function and WM can be important alterations in ASD development^34^. Research on WM and ASD has found inconsistent results since some studies have found normal WM performance in ASD (e.g.,^35,36^). However, recent findings indicate that WM deficits are especially related to both the severity of symptomatology and the functional outcomes^37–40^. Moreover, neurobiological studies reveal that, during WM tasks, subjects with ASD present deficits in brain activity in spite of not presenting clear behavioral alterations. Using fMRI studies, the neurobiological deficits have been mainly found in prefrontal areas^41–45^. Few studies assess the oscillatory activity related to WM in ASD. Only recently, in children with ASD, two interesting studies reported deficits in the evoked activity and the inter-site synchronization of the oscillatory activity^46,47^. However, the experiential design used in these studies did not allow separating deficits in the stimuli codification or in the brain network related to the maintenance of items in WM.

In this article, we tested the hypothesis that subjects with ASD present alterations in the oscillatory activity related to the neuronal computation required for WM processing. For this, we studied WM in ASD and typically developing (TD) subjects while undergoing electroencephalographic recordings. By means of time-frequency and linear modeling analyses, we assessed oscillatory modulations related to the increase of memory load. Interestingly, we found that subjects with ASD demonstrated alterations in the increases of alpha and theta oscillations associated with the increase of memory load. The theta alteration was correlated to the severity of the disorder.

## Methods

### Participants

Twenty TD subjects (8 women, mean age 21.9 ± 3.9 [s.d.]) and twenty-one ASD subjects (1 woman, mean age 21.9 ± 4.1 [s.d.]) participated in this study (see Table 1). Another ASD subjects was recruited, but was excluded from the final sample because he did not meet the full diagnosis criteria for ASD. The TD participants were 14 to 27-year-old native Spanish speakers with neither history of language impairments nor neurological/psychiatric diagnosis. They had neither sibling nor relative with suspected or diagnosed ASD. The ASD participants were 13 to 27-year-old native Spanish speakers with neither medical records of auditory processing disorder nor a syndromic disorder diagnosis. Considering the date of data collection (from 2008 to 2012), the ASD subjects were selected according to clinical evaluation by neurologists or psychiatrists following the diagnostic criteria of the Diagnostic and Statistical Manual of Mental Disorders, fourth edition^48^. Due to the updating made in the diagnostic criteria of ASD in 2013, we re-assessed each ASD evaluation under the criteria of the Diagnostic and Statistical Manual of Mental Disorders, fifth edition^49^. Following these criteria, all ASD subjects met the criteria for both persistent deficits in social communication and social interaction, and the presence of restricted, repetitive patterns of behavior, interests and  activities.

**Table 1:** Demographic and symptomatological features of samples.

	TD	ASD	*p-*value
Sample size (N)	20	21	
Demographic Data			
Participants	8 women	1 woman	p = 0.03
Mean age (years)	21.9 ± 3,9 s.d.	21.9 ± 4.1 s.d.	p = 0.73
Intellectual Mmeasures			
Average I.Q. score		96.5 ± 11.9 s.d.	
Average standard score in comprehension sub-test		7.8 ± 3.5 s.d.	
Diagnostic Measures Dsm-5 ASD symptoms (Number of ASD participants who met the full diagnostic criteria)			
Persistent deficits in social communication and social interaction (cuttoff: 3 of 3)	—	21	—
Deficits in social-emotional reciprocity	—	21	
Deficits in non-verbal communicative behaviors used for social interaction	—	21	—
Deficits in developing, maintaining, and understanding relationships	—	21	—
Restricted, repetitive patterns of behavior, interests, or activities (cuttoff: 2 of 4)	—	21	—
Stereotyped or repetitive motor movements, use of objects, or speech	—	19	—
Insistence on sameness, inflexible adherence to routines, or ritualized patterns or verbal nonverbal behavior	—	20	—
Highly restricted, fixated interests that are abnormal in intensity or focus	—	19	—
Hyper- or hyporeactivity to sensory input or unusual interests in sensory aspects of the environment	—	13	—
Symptoms are present in the early developmental period	—	21	—
Clinically significant impairment in social, occupational, or other important areas of current functioning	—	21	—
Intellectual disability	—	0	—
AMSE score (cuttoff of ≥5)			
Average score	—	8,3 ± 3,2 s.d.	—

s.d. = statistical deviation. I.Q. = intelligence quotient.

The experimental protocol and all methods were performance in accordance to institutional guidelines and were approved by the Ethical Committee of the Pontificia Universidad Católica de Chile. All participants gave their written informed consent before starting the experiment.

### Assessment

The evaluation of the ASD symptoms were subsequently revised following the observational assessment of the Autism Mental Status Exam (AMSE)^50^. This scale evaluates 8 items that can be scored 0, or 1, or 2 according to the clinician observation of social, communicative, behavioral functioning. The score obtained ranged from 0 to 14. According to the validation data^51^, a cutoff ≥ 5 predicts ASD classification on the Autism Diagnostic Observation Schedule (ADOS)^52^, which is the current golden standard observational assessment tool used in research settings in order to diagnose ASD (http://autismmentalstatusexam.com). The average AMSE score obtained in subjects with ASD participants was 8.3 ± 3.2 s.d.

In order to rule out that ASD symptoms are not due to intellectual disability (intellectual developmental disorder), the intellectual functioning of each participant was assessed. We used a cutoff < 70 points in Wechsler Intelligence Scales. Adolescents were evaluated using the Wechsler Intelligence Scale for children-revised (WISC-R)^53^ and the “Escala de Wechsler de Inteligencia para niños, tercera edición – versión chilena” (WISC-IIIv.ch)^54^. Adults were assessed using the Wechsler Adult Intelligence Scale-Revised (WAIS-R)^55^. Furthermore, social functioning was measured using the standard score obtained in the Wechsler Scale comprehension sub-test. None of the subjects with ASD met the criteria for intellectual disability. Finally, speech-language impairments were assessed by a speech therapist using the “Protocolo de evaluación pragmática del lenguaje PEP-L”^56^.

### Task

Subjects performed a modified Sternberg task^57^ while the EEG was recorded. Four-hundred milliseconds after a fixation crux placed at the center of the screen, a sequence of 1, or 3, or 5 consonants were presented (the memory set for 1, 3 and 5 memory load, respectively, 500 ms each consonant and an inter-stimulus interval of 400 ms). After a 1.7 s retention interval (blank screen), the probe replaced the fixation cross and was displayed during the first 500 ms of the recognition interval (1000 ms). Subjects were instructed to press a button as quickly as possible without making mistakes in order to indicate when the probe was a part of the memory set. The subject’s hand of response was counter-balanced across subjects. The subjects were allowed to rest and blink (relax mode) after a random number of trials. We set 45 positive trials (the probe is part of the memory set), as well as 45 negative trials (the probe is not part of the memory set) for each memory load, thus totaling 270 trials per subject. The trials were presented in blocks for each memory load, in increasing order of complexity.

### EEG recordings

Continuous EEG recordings were obtained with a 40-electrode NuAmps EEG System (Compumedics Neuroscan). All impedances were kept below 5 kΩ. Electrode impedance was retested during pauses to ensure stable values throughout the experiment. All electrodes were referenced to averaged mastoids during acquisition and the signal was digitized at 1 kHz. Electro-oculogram was obtained using four electrodes with both vertical and horizontal bipolar derivations. All recordings were acquired using Scan 4.3 (Compumedics Neuroscan) and stored for off-line treatment. At the end of each session, electrode position and head points were digitalized using a 3D tracking system (Polhemus Isotrak).

### EEG data analysis

EEG signals were preprocessed using a 0.1–100 Hz band-pass filter. Eye blinks were identified by a threshold criterion of ±100 μV, and their contribution was removed from each dataset using Independent Component Analysis (ICA). Other remaining artifacts (e.g., muscular artifacts) were detected by visual inspection of both the row-signal and the spectrogram. We thus obtained 240 ± 26 artifact-free trials per subject (ASD:228; TD:251, p = 0.006). All artifact-free trials were transformed into current source density (CSD) that was estimated using the spherical spline surface Laplacian algorithm suggested by^58^ and implemented by^59,60^. CSD computes the second spatial derivative of voltage between nearby electrode sites, acting as a high-pass spatial filter. The CSD transformation highlights local electrical activities at the expense of diminishing the representation of distal activities. Induced power distribution was computed using Wavelets transform, with a 5-cycle Morlet wavelet, in −2.7 to 1 s windows around the probe. This time-window include 0.5 seconds of inter stimulus interval, 0.5 seconds of the last stimulus of the memory set, 1.7 seconds of retention period, 0.5 seconds of the probe presentation and 0.5 second of response window. Additionally, we segmented a time-window of 1 second before the first item of the memory set (during the inter-trial interval) for the baseline normalization. For all analyses, we used the dB of power related to the baseline.

### Source Reconstruction

The neural current density time series at each elementary brain location was estimated by applying a weighted minimum norm estimate inverse solution^61^ with unconstrained dipole orientations in single-trials. A default anatomy of the Montreal Neurological Institute (MNI/Colin27) wrapped to the individual head shape (using ~300 head points per subject) was used as a brain model to estimate the current source distribution. We defined 3 × 6700 sources constrained to the segmented gray cortical volume (3 orthogonal sources at each spatial location) in order to compute a three-layer (scalp, inner skull, outer skull) boundary element conductivity model and the physical forward model^62^. The measured electrode level data $X(t)=[{x}_{1}(t),\cdots ,{x}_{n\_electrode}(t)]$ is assumed to be linearly related to a set of cortical sources $Y(t)=[{y}_{1}(t),\cdots ,{y}_{m\_source}(t)]$ and additive noise $N(t):X(t)=LY(t)+N(t)$, where L is the physical forward model. The inverse solution was then derived as $Y(t)=WX(t)=R{L}^{T}{(LR{L}^{T}+{\lambda }^{2}C)}^{-1}X(t)$ where W is the inverse operator, R and C are the source and noise covariances, respectively, the superscript T indicates the matrix transpose, and λ^2^ is the regularization parameter, R is the identity matrix that was modified in order to implement depth-weighing (weighing exponent: 0.8^63^). The regularization parameter λ was set 1/3. In order to estimate cortical activity at the cortical sources, the recorded raw EEG time series at the sensors X(t) were multiplied by the inverse operator W to yield the estimated source current Y(t) as a function of time. Thus $Y(t)=WX(t)$. Since this is a linear transformation, it does not modify the spectral content of the underlying sources. Therefore, it is possible to undertake time–frequency analyses directly on the source space. Finally, we reduced the number of sources by keeping a single source at each spatial location that pointed into the direction of maximal variance. For this, we applied a principal component analysis to covariance matrix obtained from the 3 orthogonal time series estimated at each source location. This resulted in a single filter for each spatial location that was then applied to the complex valued data in order to derive frequency specific single trial source estimates. This approach has been successfully used for our group, as well as other groups^64,65^. Since we used a small number of electrodes (40) and no individual anatomy for head model calculation, the spatial precision of the source estimations is limited. In order to minimize the possibility of erroneous results, we only present source estimations if there are both statistically significant differences at the electrode level and the differences at the source levels survive a multiple comparison correction (false discovery rate, q = 0.05).

### Statistical analysis

We used the Kolmogorov-Smirnoff test for normality. When the data did not meet the normal assumption, we used non-parametric tests. To test for differences as well as interaction between diagnosis and memory load, we used mixed-effect analysis of variance. Memory load was evaluated as within-subject factor, and diagnosis as between-subject factor. We next re-evaluated pair comparisons using Wilcoxon test and Bonferroni. To analyze linear modulation related to memory load, we used both the Mixed Linear Model.

For the EEG statistical analysis, we first fitted a general linear model (GLM) of the power of the oscillatory activity per trial in each subject (first level analysis). We thus obtained a 3D matrix of t-value (sensor, time, frequency) for each regressor and subject. We then explored for differences between groups and conditions using the Wilcoxon test (second level analysis). To correct for multiple comparisons in time-frequency charts, we used the Cluster-based Permutation test^66^. Briefly, in this method, the clusters of significant areas were defined by pooling neighboring sites (in the time-frequency chart) that showed the same effect (uncorrected p < 0.05). The cluster-level statistics was computed as the sum of the statistics of all sites within the corresponding cluster (e.g., Z value for Wilcoxon test). We evaluated the cluster-level significance under the permutation distribution of the cluster that had the largest cluster-level statistics. The permutation distribution was obtained by randomly permuting the original data (i.e., permuting memory load label per trial for within subject analyses, or group label (ASD or TD) for between subject analyses). After each permutation, the original statistics test was computed (i.e., Wilcoxon test), and the cluster-level statistics of the largest resulting cluster was used for the permutation distribution. After 1000 permutations, the cluster-level significance for each observed cluster was estimated as the proportion of elements of the permutation distribution larger than the cluster-level statistics of the corresponding cluster.

### Software

All behavioral statistical analyses were performed in R. The EEG signal processing was implemented in MATLAB using CSD toolbox^59^, in-house scripts (available online http://neurocics.udd.cl), BrainStorm^67^ and openMEEG toolboxes^68^.

## Results

### Behavior

**Figure 1:**
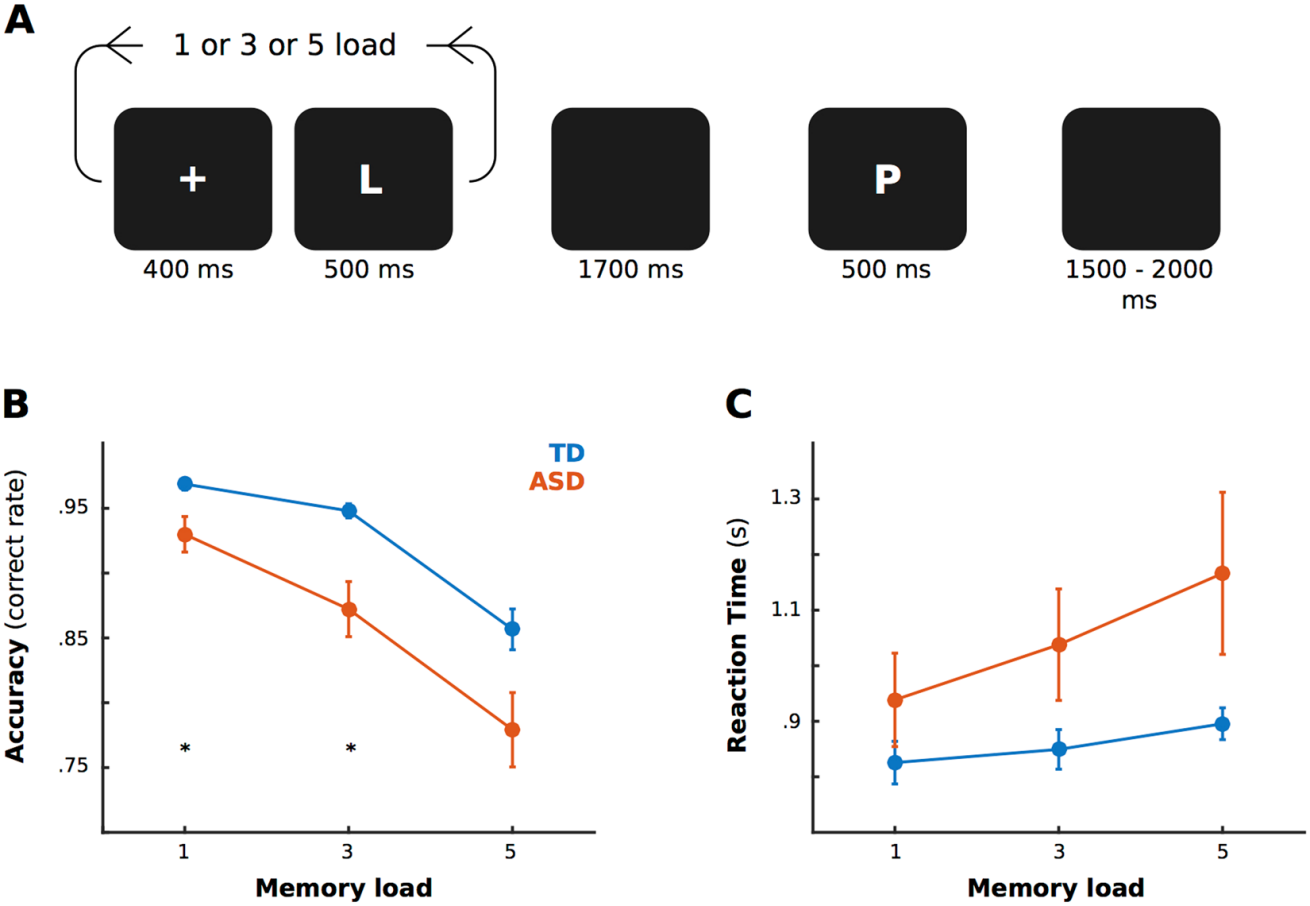
Task and Behavioral results. (**A**) Timeline of a trial during the task. (**B**) Accuracy separated by memory load and groups. (**C**) Reaction time separated by memory load and groups. (**B**,**C**) Blue lines represent the typically developing (TD) group and red lines the Autism Spectrum Disorder (ASD) group. Error bar indicated Standard error of means, and asterisks significant differences between groups (p < 0.05, Wilcoxon and Bonferroni correction).

Both groups of subjects presented a decrease in the accuracy related to the increase in memory load (see Fig. 1, mixed ANOVA, Factor Load, F = 8.6, p = 3.15e-4), though in general ASD presented greater error (Facto Diagnosis, F = 22.9, p = 4.8e-6, Interaction F = 2.1, p = 0.1). The difference between the groups is significant for memory loads 1 and 3 (Wilcoxon Test and Bonferroni Corrected, Load 1, p = 0.01, Load 3, p = 0.02, load 5 p = 0.2). We evaluated linear decrease in accuracy using a mixed linear model. This analysis revealed a significant difference between the groups (Memory Load: T = −5.6, p = 1.1e-7; interaction Memory Load*Diagnosis: T = −2.1, p = 0.045). In order to control for possible non-linear decrease in the accuracy, we tried several models using quadratic factors. The best fitted model included a quadratic factor for Memory Load. Interestingly, this model improves the general adjustment, as well as the significance of the interaction between Memory Load and Diagnosis (see Supplementary Table 1). Overall, these results suggest that subjects with ASD presented not only an overall decrease in their accuracy, but also a greater decrease in the accuracy related to the memory load increase.

For the reaction time (taking into account only correct responses), subjects with ASD presented greater values (Diagnosis factor, F = 21.8, p = 7.3e-6). Neither group presented an increase in their reaction time related to the memory load increase (Load factor, F = 1.0, p = 0.3; Interaction: F = 0.4, p = 0.6). The mixed linear analysis did not exhibit significant differences between the groups.

### Oscillatory activity

Following prior work, we explored parametric power modulation of oscillatory activity related to the memory load. For this, we segmented the signal in order to study the coding of the last stimulus of the memory set and the retention period. Using a general linear model for single trial time-frequency chart (see Methods), we explored significant modulation related to memory load within each group of subjects and between groups. During the estimation of the individual regressor per subject (first level analysis), we included erroneous responses as an independent regressor to rule out differences in the oscillatory activity given only by accuracy differences.

**Figure 2:**
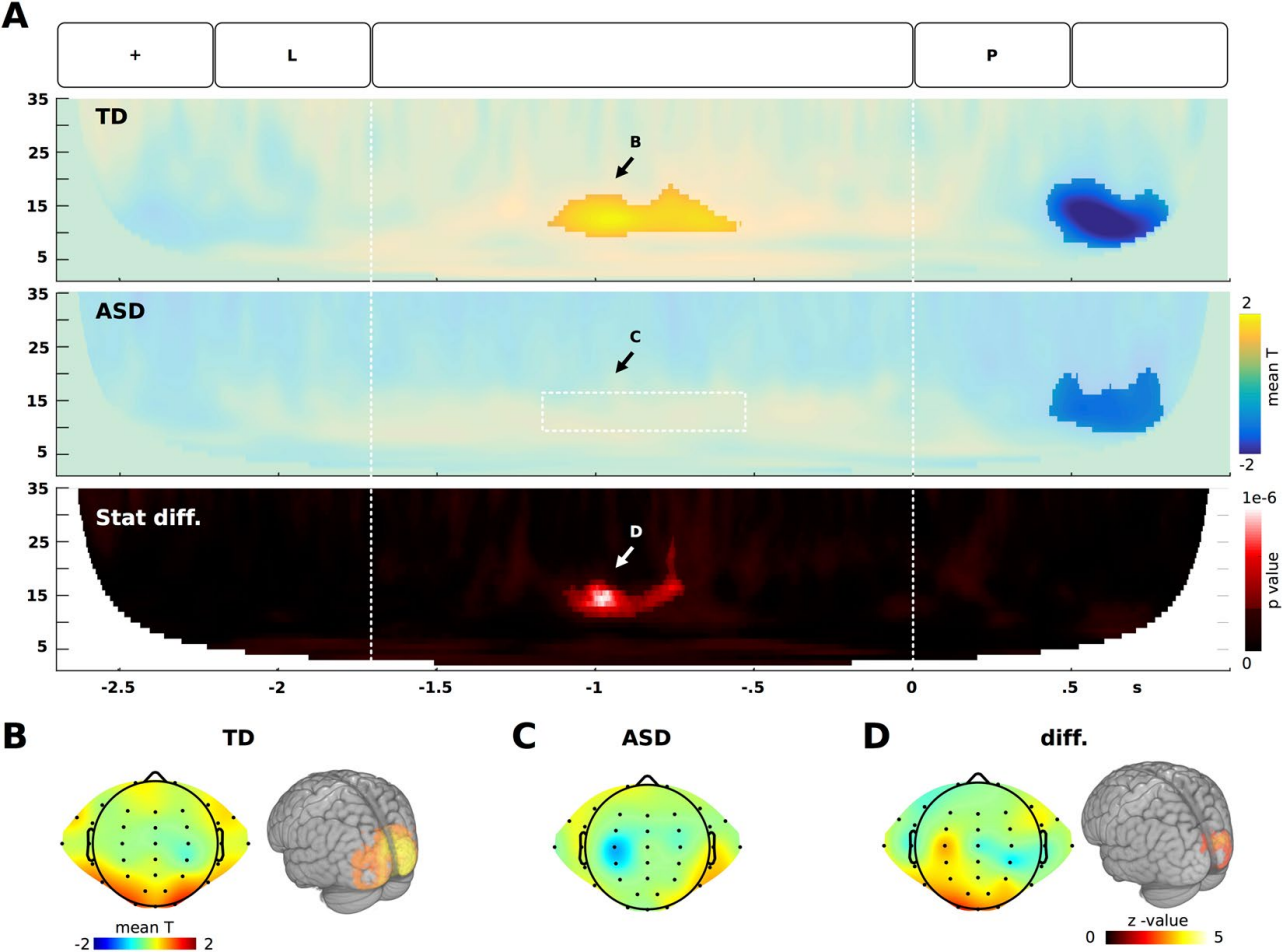
Alpha oscillation increase related to memory load. (**A)** Time-frequency modulation in the occipital electrodes. In the superior and middle panels, colors represent the mean across subjects of the t-value of individual correlations between the power of the oscillatory brain activity and the memory load. In the inferior panel, colors represent the p-value of the between group differences. The clusters with significant effects are highlighted (p < 0.01, cluster-corrected). (**B–D**) Scalp distribution and source estimation of alpha modulation as indicated in **A**.

In accordance to prior reports^69–72^, we found modulations in alpha and theta bands in the control group. For alpha band, we found a positive modulation between the power of bilateral occipital electrodes and the memory load (see Fig. 2). This modulation was placed between 9 to 15 Hz, obtaining its maximum modulation at 0.7–1.2 s during the retention period. In the source space, this modulation was placed at the occipital cortex. This placement is in accordance with previous reports which used both source reconstruction^26^ and BOLD-EEG correlation^29^, indicating that this activity probably reflects a decrease in neuronal activity. By contrast, the ASD group did not demonstrate modulation in alpha band. Therefore, during the retention period, we found a significant difference between the groups in alpha band in the occipital electrodes. The sources of this difference were located at the occipital cortex.

**Figure 3:**
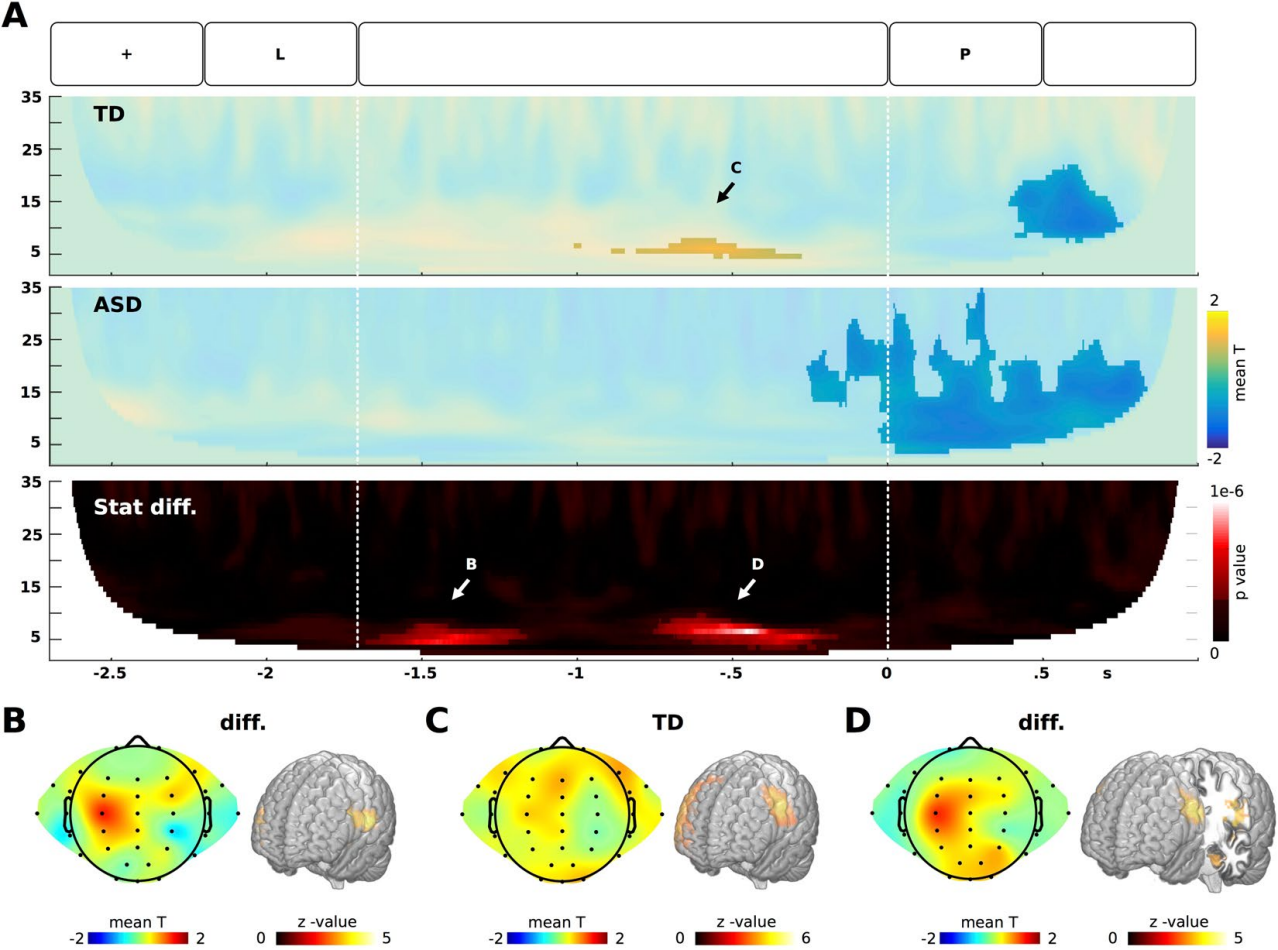
Theta oscillation increase related to memory load. (**A**) Time-frequency modulation in the left-central electrodes. In the superior and middle panels, colors represent the mean across subjects of the t-value of individual correlations between the power of the oscillatory brain activity and the memory load. In the inferior panel, colors represent the p-value of the between group differences. The clusters with significant effects are highlighted (p < 0.01, cluster-corrected). (**B–D**) Scalp distribution and source estimation of the theta modulation as indicated in (**A**).

We also found a modulation in theta band related to the memory load. In the TD group, we found an increase in theta band (5 to 8 Hz) that correlated with the memory load (see Fig. 3). The modulation reached significance during the last part of the retention period in the frontal and left lateralized central electrodes. In the source space, this modulation was placed at both the posterior part of the inferior/middle frontal gyros (premotor cortex) in the left hemisphere and the anterior part of the middle frontal gyros in the right hemisphere. The ASD group did not demonstrate significant modulation in theta band, showing significant differences with the TD group. These differences took place at the beginning and at the end of the retention period. In both cases, the differences were placed at the left fronto-central electrodes and their sources were placed mainly at the premotor cortex.

Finally, we assessed for possible correlations between the impairments in the oscillatory modulations and the symptomatology of the ASD group. For this, we used the AMSE score and the individual regressor for the modulation of alpha and theta bands placed on the maximum difference between the groups in the scalp. We found that, for theta band modulation, there existed a negative correlation with the symptomatology (rho = −0.42, p = 0.04, C3 electrode). This indicated that patients with less theta increase presented more autistic symptoms. For alpha band, we found the same trend though it was not significant (rho = −0.27, p = 0.2, O2 electrode).

### Control analyses

Although, in the ASD group, the linear regression of behavioral data indicated a greater decrease in the accuracy across conditions, we did not find significant differences for memory load 5. This can be interpreted as a general sensory deficit, though not a specific deficit in memory load. In order to demonstrate the specificity of the oscillatory activity deficit to the memory load, we carried out two control analyses. In one of the preceding, we compared the event-related potentials, as well as the oscillatory activity related to the encoding of the item of memory set in the condition load 1. Interestingly, we did not find any differences in the sensory components P1 and N1 between the groups (see Supplementary Figure 1). Similarly, no clusters of oscillatory activity in the occipital electrodes survived multiple comparison corrections (see Supplementary Figure 1). In the other analysis, we carried out a categorical comparison between two memory load conditions. We selected different conditions per group in order to compare conditions with similar accuracy between the groups. Thus, we tried to control for possible differences related to the degree of difficulty and not related to the pathology per se. For the ASD group, we used memory loads 1 and 3, and for the TD group, we used memory loads 3 and 5 (ASD accuracy of memory load 1 vs TD accuracy of memory load 3: uncorrected p = 0.8; ASD accuracy of memory load 3 vs TD accuracy of memory load 5: uncorrected p = 0.2). In this analysis, we found no differences between the groups for alpha activity (see Supplementary Figure 2). Interestingly, for theta activity, we replicated the differences found in the left parietal electrode (see Supplementary Figure 3). The preceding reveals that the differences in theta activity could not be attributed only to a difference in task difficulty.

## Discussion

The aim of this study was to assess possible oscillatory brain activity impairments related to cognitive computations in ASD. For this, we used a well-known sequential verbal WM task, namely the Sternberg task. Solving this kind of WM task requires the retention of verbal information in the absence of external stimuli. This process involves a distributed brain network. This network, among others, includes the prefrontal and posterior-parietal cortices, as well as the hippocampal formation and basal ganglia^73,74^. In this context, a crucial issue concerns how neuronal assemblies represent and sustain information in the absence of sensory inputs. Current evidence indicates that effective information transmission across neural structures requires the spatiotemporal coordination of oscillatory activity. This being so, reverberating neuronal activity in distributed cell assemblies may underlies information maintenance during WM^75^. In fact, recent experimental and theoretical approaches point out that hierarchical oscillatory organization between low (theta, alpha) and high (beta, gamma) frequencies facilitates the formation of coherently organized groups of neurons via the establishment of transient temporal correlated activity^31,76,77^. Crucially, animal model evidence indicates that an imbalance in neural excitatory and inhibitory activities may be related to ASD pathogenesis^8,25,78^. For example, studies suggest that GABAergic interneurons are related to the etiology of ASD^6,78^. This inhibitory interneuron system is closely related to rhythmogenesis, which is necessary for the generation of cortical network activity patterns during cognitive processing, especially WM^5^. Thus, possible alterations in the oscillatory activity during WM in ASD can be a good marker of the rhythmogenesis alteration in these patients.

In accordance with the literature, in TD subjects, we found that the memory load modulated the amplitude of different frequencies, in particular theta and alpha bands. An increase in alpha power during retention period is the most consistently reported modulation in scalp recordings^26,27,72,79–82^, although the functional interpretation is conflicting. Alpha oscillations probably participate in both inhibit task-irrelevant cortical areas and generate a pacemaker to filter or temporality select relevant information from sensory cortices^26,79,80,83–85^. As expected, we found that TD subjects presented an alpha load-modulation located at the visual cortex and that subjects with ASD did no. Interestingly, to rule out difficulty differences, we did not find any differences in alpha band between the ASD and TD groups. This may reflect that the activity related to the inhibition of task-irrelevant cortical areas is closely related to task difficulty, and may not necessarily reflect an inherent alteration of ASD.

Our findings complement and deepens recent research which use the n-back task in ASD children. Since our experimental design allows us to separate encoding, retention, and retrieval of WM, we demonstrated that subjects with ASD presented a specific impairment during the retention of items in WM. Consequently, theta impairments can reflect an alteration in the temporal organization of WM items. Indeed, computational simulations and intracranial recording in human have demonstrated that theta gamma phase coupling may provide a mechanism by which multiple item representations can be sequentially activated in each theta cycle^76,86–88^. Although the representation of deep structures in the scalp EEG is poor, our source analyses reveal that the hippocampus can participate in the theta modulation together with lateral frontal cortex (see Fig. 3D). Interestingly, using both MEG and intracortical recordings in humans, studies have demonstrated that the theta/gamma modulation in the hippocampus is crucial for the item-context binding during episodic memory^86,87^. Lesion studies have found that the hippocampus is also important in WM^89,90^. Thus, theta activity could be particularly relevant to maintenance of temporal sequence information^91,92^. Interestingly, theta oscillations are closely related to inhibitory interneurons system^93^. Thus, theta oscillations may reflect oscillating levels of inhibition that bias the competition between representations in a network generating a selection of relevant information during the retention period^91,92^.

Concerning ASD, other studies that used during a WM task found that these patients did not present a modulation in the ERP in the hippocampus as compared to healthy children^47^. ASD patients present structural abnormalities in the hippocampus^94,95^. Additionally, decreases in the hippocampus connectivity are related to episodic memory alterations in adults with ASD^96^. Interestingly, this hypoconnectivity includes areas in left and right lateral prefrontal regions where we also found alterations in theta band. In ASD, alterations in the left lateral PFC BOLD signal have been related to memory encoding and successful retrieval^96,97^. During WM tasks, similar reductions have been found in ASD patients^41,43^ However, due to the temporal limitation of the fMRI technique and the design of working memory tasks (e.g., n-back) it is not possible to identify the specific altered process in ASD. In this context, taking the preceding evidence into account, our findings give a new insight on memory alteration in ASD. We found that while TD subjects showed an increase of the power of theta activity related to memory load, subjects with ASD did not show any increases. This difference remains significant to control for the difficulty of the task. This pattern of oscillatory alterations might indicate that a key feature of these patients is the impairment to recruit adequate oscillatory activity in accordance with the computational demand. The preceding reveals that subjects with ASD present an alteration in the capacity to maintain and probably to manipulate information online during WM. The neurobiological mechanism underlying this alteration seems to be related to the incapacity to recruit oscillatory activities according to the memory load demand.

In summary, the oscillatory activity in theta range is known to enable efficient transmission and coding of information in distributed neuronal populations^5,98^. That being so, the oscillatory alterations in ASD include a network of well-known regions participating in memory processing, namely the lateral prefrontal cortex and the medial temporal region. Indeed, we found that theta alteration correlated with the autistic symptomatology. While it is true that the alterations that we found are related to WM, oscillatory impairments can be a key step into the physiopathology of autism.

## Electronic supplementary material

Supplementary material
