# Supplementary material for: Theta and Alpha Oscillation Impairments in Autistic Spectrum Disorder Reflect Working Memory Deficit
Source: Sci Rep. 2017 Oct 30;7:14328. doi: 10.1038/s41598-017-14744-8 (PMC5662653; doi:10.1038/s41598-017-14744-8)
Supplement: Supplementary file 1 — Supplementary material [file 41598_2017_14744_MOESM1_ESM.pdf]

Supplementary material

Theta and Alpha Oscillation Impairments in Autistic Spectrum Disorder Reflect Working Memory Deficit

Josefina Larrain-Valenzuela[1#], Francisco Zamorano[1,2#], Patricia Soto-Icaza[3], Ximena Carrasco[3], Claudia Herrera4, Francisca Daiber[3], Francisco Aboitiz[3], Pablo Billeke[1]*.

1 División de Neurociencia, Centro de Investigación en Complejidad Social (neuroCICS), Universidad del Desarrollo, Av. Las Condes 12461, Las Condes, Santiago, Chile 7590943

2 Unidad de Imágenes Cuantitativas Avanzadas, Clínica Alemana de Santiago, Av. Vitacura 5951, Vitacura 7650568, Chile

3 Laboratorio de Neurociencias Cognitivas, Departamento de Psiquiatría, Centro Interdisciplinario de Neurociencia, Pontificia Universidad Católica de Chile, Marcoleta 391, Santiago 8330024, Chile

4 Sociedad de Psiquiatría y Neurología de la Infancia y Adolescencia de Chile, Esmeralda 678, Santiago 8320053, Chile.

* Address correspondence to Pablo Billeke MD., PhD., División de Neurociencia, Centro de Investigación en Complejidad Social (neuroCICS), Facultad de Gobierno, Universidad del Desarrollo. Av. Las Condes 12461, Las Condes, Santiago, Chile. Email: pbilleke@udd.cl

# Equal contribution

Supplementary Table 1: Linear mixed model of Accuracy

	slope	Standard error	t-value	p-value
Intercept	-0.09	0.07	-1.3	0.19
Conditions	0.1	0.07	1.3	0.18
Conditions^2	-0.04	0.01	-2.57	0.011
Diagnosis (ASD)	-0.02	0.05	-0.42	0.67
Conditions*Diagnosis	-0.04	0.02	-2.08	0.038

Degree of freedom: 124; AIC: -151.69; BIC: -131.67; Log-Likelihood: 82.846; Deviance: -165.69

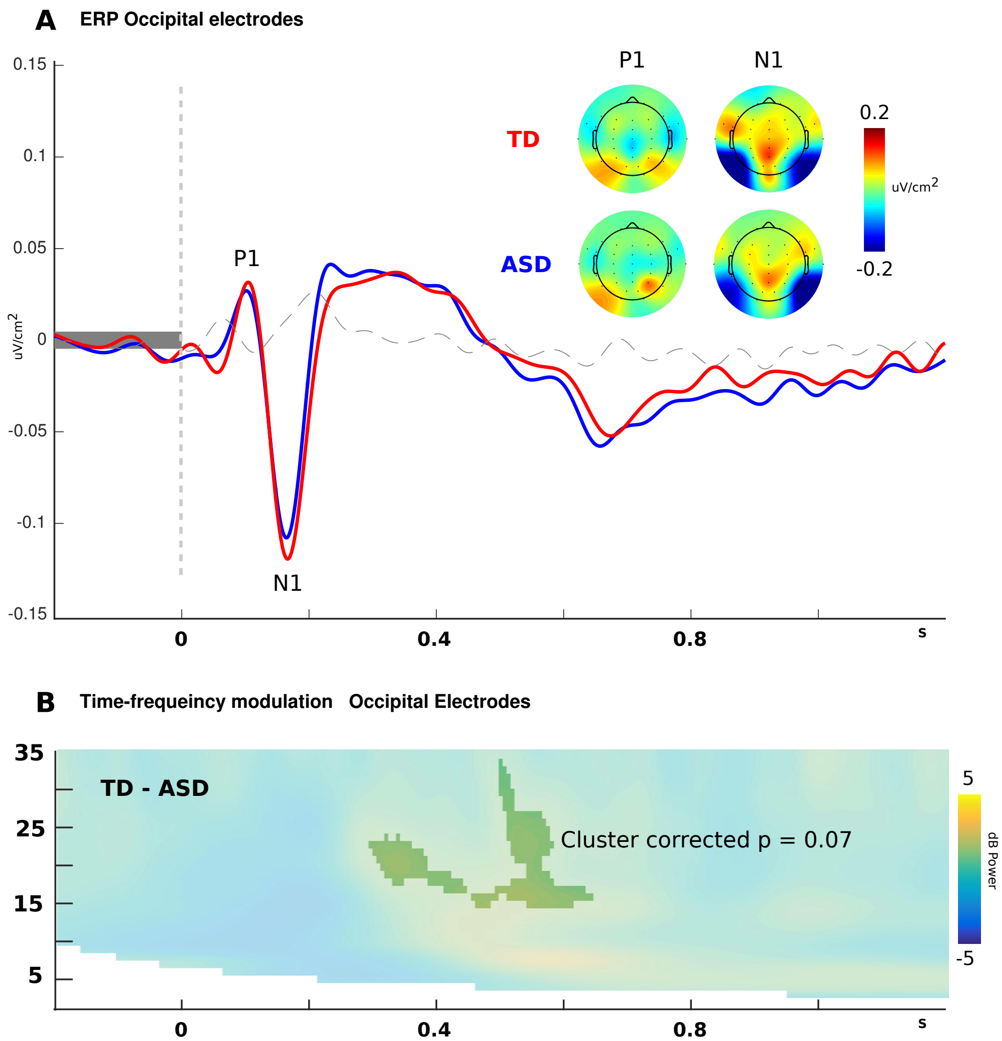

Supplementary Figure 1: Encoding. **A** ERP analysis of the occipital electrodes for the first item of the memory set in condition load 1 for both groups. The red line represents the typically developing (TD) group and the blue line the autistic spectrum disorder (ASD) group. No significant differences were found at uncorrected threshold (p<0.05, k=10). **B** Time frequency analysis. Color represents the difference between groups. One cluster was found with a cluster-corrected p value of 0.07.

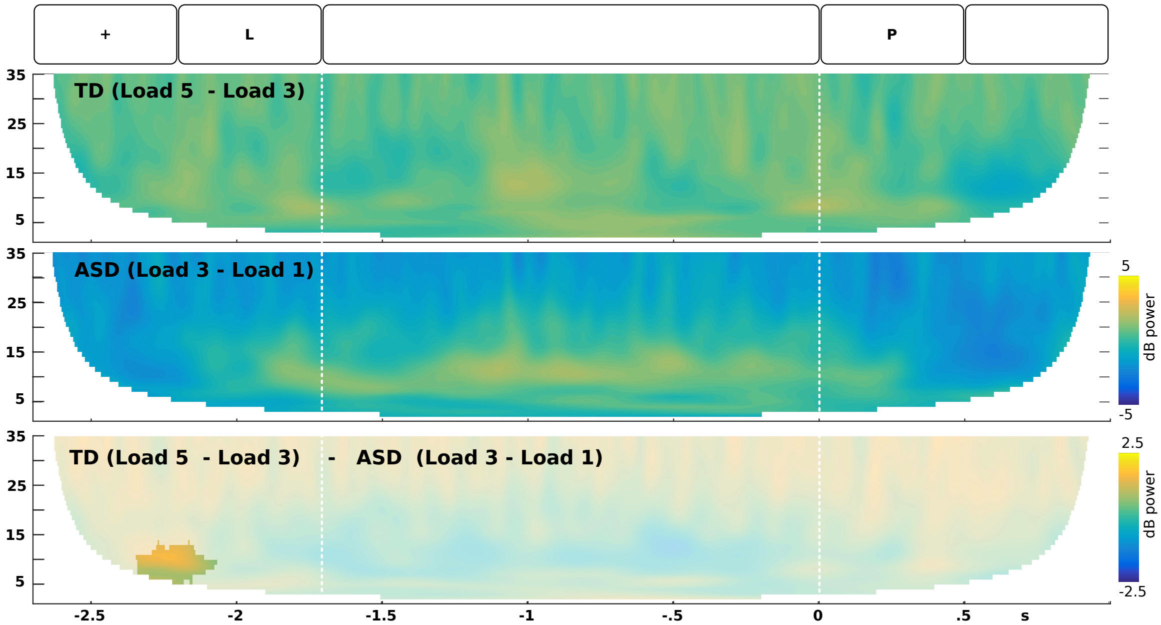

Supplementary Figure 2. Categorical analysis for the increase in memory load in conditions with comparable accuracy per group in the occipital electrodes (as shown in Figure 2). Note that no cluster survived multiple comparison correction during retention period.

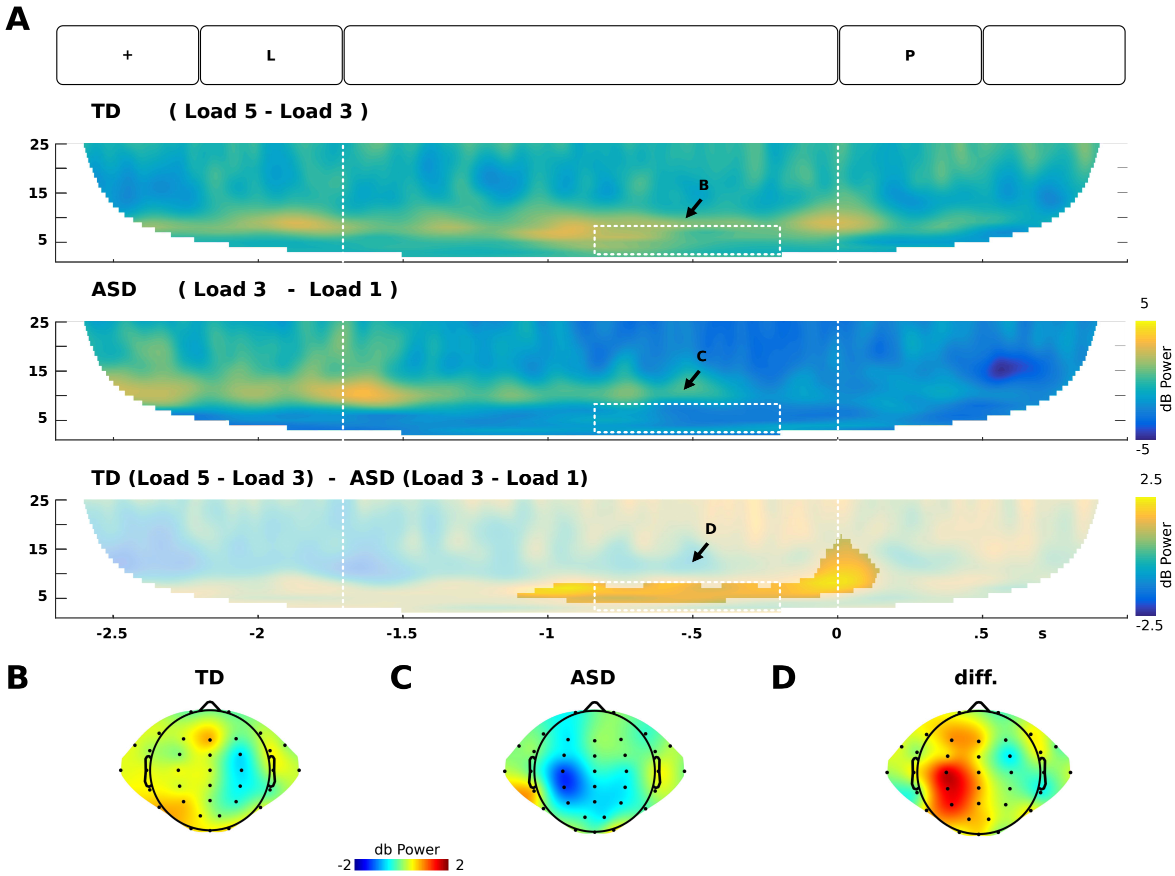

Supplementary Figure 3. Categorical analysis for the increase in memory load in conditions with comparable accuracy per group in left frontal electrodes (as shown in Figure 3). The scalp distributions shown in B, C, and D were extracted from the same time-frequency windows as those of Figure 3. Note that we found a significant modulation in theta activity.
